# Migration-Associated Transportome and Therapeutic Potential in Glioblastoma Multiforme (GBM)

**DOI:** 10.3390/cancers15133514

**Published:** 2023-07-06

**Authors:** Samir Vaid, Mirko H. H. Schmidt

**Affiliations:** Institute of Anatomy, Medical Faculty Carl Gustav Carus, Technische Universität Dresden School of Medicine, Fetscherstr 74, 01307 Dresden, Germany

## 1. Introduction

GBM is a highly aggressive and very common malignant form of primary brain tumors in adults [1,2]. It is characterized by its infiltrative nature, rapid growth, and resistance to treatment, making it a formidable challenge in the field of oncology [3]. There is a paucity of understanding on the etiology of GBM because, in most cases, GBM occurs sporadically without a clear identifier.

The treatment of GBM involves a multidisciplinary approach, combining surgery, radiation therapy, and chemotherapy [4,5]. Despite aggressive treatment, the prognosis for GBM remains poor, with a median survival of around 15 months [6,7,8]. Of the many challenges associated with the treatment of GBM, cell migration poses a significant bottleneck in the treatment of this cancer. GBM cells are highly invasive and have the ability to infiltrate surrounding healthy brain tissue. They can extend microscopic tendrils into neighboring regions of the brain, making it extremely difficult to completely remove the cancerous cells during surgery [9]. Additionally, even when the main tumor mass is removed, the infiltrated cells often remain, inevitably leading to tumor recurrence, which is related to shorter survival chances [10]. In the majority of recurring cases, GBM recurs locally (75–80%) [11,12,13], but the relapses can also occur in anatomically distant regions, as far as the contralateral hemisphere (4%) [13]. GBM cells can also evade the blood–brain barrier, allowing them to migrate beyond the primary tumor site and evade treatment [14]. GBM is a highly heterogeneous cancer, meaning that it consists of a diverse population of cancer cells with different genetic and molecular characteristics. Some subpopulations of cells have been shown to have increased migratory capabilities [9,15,16,17,18]. This heterogeneity likely contributes to the adaptability and resistance of GBM cells, making it challenging to target and eliminate all migrating cells effectively. Therefore, to enhance treatment options for GBM, there is a continuous exploration for innovative strategies to target such migrating cells.

The solute carrier proteins (SLCs) are membrane-bound proteins that maintain cellular homeostasis by facilitating the exchange of a broad range of substrates, including nutrients, ions, neurotransmitters, and drugs [19]. The SLCs, therefore, are involved in many biological processes, including cell migration. Dysregulation of SLCs can disrupt these processes and lead to impaired cell migration, which can have significant implications including cancer progression [19,20]. To advance understanding of the genetic programs regulating cell migration in GBM cells, the study by Brosch P.K. et al. [21] specifically focused on the role of two SLCs, SLC5A1 and SLC5A3, and their respective substrates, glucose and inositol [21].

Using cell migration assay [22], the authors found that both glucose and inositol significantly increased the cell migration in two highly motile glioblastoma cell lines, SNB19 and DK-MG [23,24]. The authors also showed that inhibiting SLC5A1 and SLC5A3 with the specific inhibitor phlorizin [25,26] significantly reduced the migration of cells in both glioblastoma cell lines. Importantly, they showed that inhibitory effect of phlorizin on the cell migration in both cell lines persisted even in the presence of supplemental solutes, glucose or inositol, demonstrating that SLC5A1 and SLC5A3 are essential SLCs for cell migration in glioblastoma cells. Consistent with their role in promoting cell migration, in both DK-MG and SNB19 cell lines, SLC5A1 and SLC5A3 protein expression was predominantly present in the motile cells facing the migration front, with SLC5A3 expression being more confined to the leading cell edge when compared to SLC5A1. The expression of both these proteins was virtually absent in the non-motile cells that were present in the regions distal to the migration front.

To gain insights into how SLC5A1 and SLC5A3 could be involved in the mobility of GBM cells, the authors compared the localization of these proteins in single cells. They observed the expression of these transporter proteins in lamellipodial tips and spike-like membrane protrusions of the cells. SLC5A1 was present in nascent expanding blebs, while SLC5A3 was exclusively localized to mature retracting blebs. Interestingly, the authors found the two transporter proteins to be co-localized in smaller retracting blebs but not in large anterior nascent blebs. SLC5A1 was also found to co-localize with tubulin in large nascent blebs of DK-MG cells. However, SLC5A3 was confined to mature blebs and did not show co-localization with tubulin.

The study then characterized if SLC5A1 and SLC5A3 are associated with the membrane permeability of GBM cells to the respective transporter substrates. The authors recorded osmotic cell swelling in hypotonic solutions containing different sugar solutes. When the GBM cells were exposed to the hypotonic solutions, regardless of the sugar used, they rapidly swelled due to water entering the cells driven by the osmotic gradient. However, there were differences in the subsequent volume changes depending on the type of sugar. When exposed to a disaccharide sugar, sucrose, after the initial swelling, the cells underwent a process called regulatory volume decrease (RVD), where they gradually shrank and returned to their original size despite the hypotonic environment. In contrast, when the cells were exposed to monomeric sugars like glucose, mannitol, and inositol, they either partially (mannitol) or completely (glucose and inositol) inhibited RVD. In SNB19 cell line, these sugars even caused secondary swelling. The authors also found differences in the two cell lines in regard to the sugar solute to which they are more permeable. The study found that glucose had the highest permeability among the tested solutes in SNB19 cells, while in DK-MG cells, inositol and glucose displayed the highest permeability. Taken together the results therefore show that GBM cell membranes are highly permeable, and the presence of specific sugars can affect the regulatory mechanisms that control cell volume of GBM cells. Whether this increased permeability is due to the high expression of SLC5A1 and SLC5A3 in the GBM cells, however, remains an open question.

### Potential Impact of the Study

Taken together, the findings suggest a synergy between two SLCs, SLC5A1 and SLC5A3, in promoting GBM cell migration. Finding SLC5A1 expression in the bleb membrane suggests that, similar to aquaporins [27,28], it might play a role in the uptake of extracellular glucose, leading to an influx of water and an increase in local volume, which is essential for bleb expansion. On the other hand, the presence of SLC5A3 mainly in smaller retracting blebs suggests that it may contribute to bleb retraction by facilitating the efflux of inositol and water, leading to a decrease in bleb volume, a role consistent with an earlier finding [29]. The study also observed that SLC transporters, including SLC5A3, can be transported to the cell membrane from cytosolic vesicles during swelling-activated exocytosis. The vesicles carrying the transporters may move along microtubules, which are protein structures within the cell. Microtubules extending into the blebs suggest that other SLC transporters may also be incorporated into blebs through vesicle transport along microtubules, and targeting this structure could be a therapeutic step to inhibit cell migration and invasion of GBM.

Efficient volume regulatory mechanisms are essential for cell survival because they protect cells against excessive osmotic shrinkage or swelling. Given that both glucose and inositol are abundant in the brain and these solutes very strongly affect the regulatory mechanisms that control cell volume of GBM cells, the study provides a therapeutic potential to exploit the SLCs, and/or other migration associated transporters, in GBM treatment.
